# The clinical effects of digital cognitive behavioral therapy for insomnia in a heterogenous study sample: results from a randomized controlled trial

**DOI:** 10.1093/sleep/zsad184

**Published:** 2023-07-10

**Authors:** Jennifer Schuffelen, Leonie F Maurer, Noah Lorenz, Alexander Rötger, Reinhard Pietrowsky, Annika Gieselmann

**Affiliations:** Heinrich Heine University Düsseldorf, Institute of Experimental Psychology, Department of Clinical Psychology, GermanyGermany; mementor DE GmbH, Department of Science, Germany; mementor DE GmbH, Department of Science, Germany; mementor DE GmbH, Department of Science, Germany; Heinrich Heine University Düsseldorf, Institute of Experimental Psychology, Department of Clinical Psychology, GermanyGermany; Heinrich Heine University Düsseldorf, Institute of Experimental Psychology, Department of Clinical Psychology, GermanyGermany

**Keywords:** cognitive behavioral therapy for insomnia, CBT-I, insomnia, sleep, digital therapy, dCBT-I, digital health, regular care, randomized controlled trial, RCT

## Abstract

**Study Objectives:**

Numerous studies worldwide have reported the beneficial effects of digital cognitive behavioral therapy for insomnia (dCBT-I). However, few focus on *real-world* study samples that reflect people in regular care. To test whether dCBT-I is suitable within German regular care, we designed a randomized controlled trial recruiting a heterogenous insomnia population.

**Methods:**

Participants aged ≥18 who met the criteria for insomnia disorder were randomized to 8-weeks dCBT-I + care-as-usual (CAU) or they were set on a waitlist + CAU. The intervention group was followed-up at 6- and 12-months. The primary outcome was self-reported insomnia severity, assessed with the Insomnia Severity Index (ISI) at 8-weeks post-randomization. A one-way ANCOVA with baseline score as a covariate was fitted to determine group differences. Secondary outcomes included measures of daytime functioning, quality of life, depression, anxiety, dreams, and nightmares.

**Results:**

Of the *N* = 238 participants (67.6% female), age range 19–81 years, *n *= 118 were randomized to dCBT-I and *n* = 120 to the control group. At posttreatment, the use of dCBT-I was associated with a large reduction in the ISI (Diff_adj_ = –7.60) in comparison to WLC (*d *= –2.08). This clinical improvement was also reflected in responder and remission rates. Treatment effects were also observed for daytime functioning, quality of life, symptoms of depression and anxiety (*ds *= 0.26–1.02) and at long-term follow-up (intervention group only; *ds *= 0.18–1.65). No effects were found for dream and nightmare frequency.

**Conclusions:**

This study showed that dCBT-I reduces insomnia symptoms and improves daytime functioning in a heterogenous insomnia population in Germany with sustained long-term treatment effects in the intervention group. Our results underscore the potential of digital health applications, their suitability within regular care, and their role in facilitating widespread implementation of CBT-I as a first-line treatment for insomnia.

## Graphical Abstract

**Figure:**
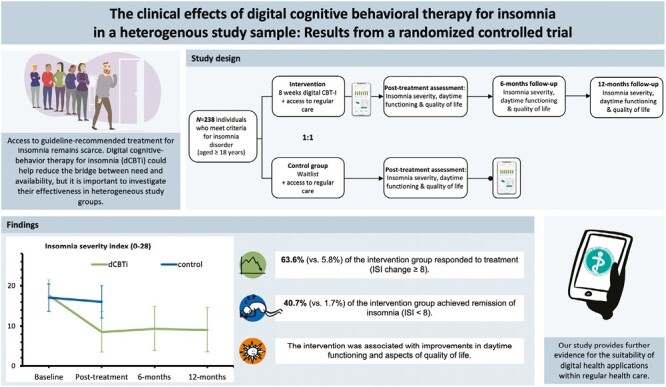

**Statement of Significance**

In this randomized-controlled trial, we tested whether digital implementation of cognitive-behavioral therapy for insomnia is an effective treatment in an insomnia population that was not limited by extensive exclusion criteria. Consequently, our recruitment yielded a study sample that was characterised by moderate-to-severe insomnia severity and high prevalence rates of comorbid physical and mental conditions. Clinical evaluation of primary and secondary study outcomes showed robust treatment effects in comparison to a waitlist control group and supports its current use within regular insomnia care. Treatment effects in the intervention group were sustained at long-term follow-up.

## Introduction

Insomnia is one of the most frequent sleep disorders and one of the most prevalent mental disorders in Europe, affecting approximately 6%–10% of the adult population [1]. It is a burdensome condition, degrades the quality of life [2, 3] and confers the risk of developing other mental health disorders [4]. Frequent co-occurring mental conditions are depression and anxiety disorders [5]. European guidelines recommend cognitive-behavioral therapy for insomnia (CBT-I) as the first-line treatment [6], which has demonstrated large and enduring treatment effects [7–9]. Still, the majority of patients with insomnia do not receive CBT-I [6, 10], most likely because dedicated care for such a large population in the usual one-to-one relationship between therapist and patient is not achievable [11]. The use of digital, Internet-based CBT-I (dCBT-I) could be a promising solution to facilitate widespread dissemination and implementation of first-line recommended treatment for insomnia [12]. Typically, dCBT-I incorporates the same components as face-to-face CBT-I and is delivered through websites or mobile apps, which include informative texts, graphs, videos, and illustrations. Some include individual tailoring through patient feedback via questionnaires and intelligent sleep diaries [13]. Digital health programmes are available to patients at flexible times and places, allowing widespread access, even to patients living in structurally weak rural regions [14]. Additionally, these interventions can bypass long waiting times whenever there is a shortage of available resources and thereby reaching patients in earlier stages of the disorder [12]. While meta-analyses have shown that treatment effects of dCBT-I are comparable to those of face-to-face CBT-I [15–17], results of direct comparisons are inconsistent [18, 19] and there is only a limited number of such trials.

In October 2020, digital therapy was introduced to the German healthcare system in the form of so-called digital health applications (German: “digitale Gesundheitsanwendungen,” DiGA) and can since be prescribed by practitioners and psychotherapists to people affected, with costs being covered by the statutory health insurance system. With regard to insomnia, little is known about the feasibility and effects of dCBT-I in pragmatic study settings in Germany. However, emerging evidence from real-world studies and large-scale RCTs across different countries demonstrates promising results in the short [20–25] and long-term [21, 26–28].

In this study, we compared dCBT-I as an add-on to care as usual (CAU) to a waitlist control group (WLC + CAU) while limiting exclusion criteria to an absolute minimum. With the unique situation of digital care in Germany in mind, the following primary and secondary clinical hypotheses were tested:

1. dCBT-I + CAU reduces self-reported insomnia severity relative to WLC + CAU.
2. dCBT-I + CAU reduces fatigue and daytime sleepiness relative to WLC + CAU.
3. dCBT-I + CAU reduce dysfunctional beliefs and attitudes about sleep relative to WLC + CAU.
4. dCBT-I + CAU improves well-being and quality of life relative to WLC + CAU.
5. dCBT-I + CAU reduces depressive and anxiety symptoms relative to WLC + CAU.
6. dCBT-I + CAU reduces dream recall frequency relative to WLC + CAU.
7. dCBT-I + CAU reduces nightmare frequency relative to WLC + CAU.

Additionally, we investigated whether potential improvements are stable over time by comparing follow-up data at 6- and 12-months follow-up in the intervention group and conducted explorative analysis on application-reported sleep diary variables.

## Methods

### Study design

The trial is a parallel-group, two-armed open-label randomized controlled trial, whereby participants were randomized to the intervention group or to the waitlist control group (WLC). The intervention group received digital cognitive behavioral therapy for insomnia (dCBT-I, *somnio*, mementor DE GmbH) over a period of 8-weeks. The WLC group received no intervention from the study team during this period but was given access to dCBT-I upon completion of the posttreatment assessment at 8-weeks post-randomization. Both groups continued to have access to care as usual (CAU). Assessments took place before the start of the intervention (baseline) and 8-weeks post-randomization (post-intervention). Additionally, the intervention group was followed-up at 6- and 12-months post-randomization. The trial was conducted in Germany, approved by the Ethics Committee of Heinrich Heine University Duesseldorf, and preregistered on February 11, 2021, at the German Clinical Trials Register (Deutsches Register Klinischer Studien; DRKS) under DRKS00024477. The study protocol can be requested from the corresponding author.

### Participants

Participants were recruited between February 23, 2021 and May 21, 2021, mostly through mailouts and online advertisement of a German health insurance company (Techniker Krankenkasse, TKK), but also through flyers in medical practices and online advertisement (social media). Eligibility requirements were: (a) a diagnosis of chronic insomnia disorder according to the DSM-5 classification criteria [29] and (b) a minimum age of 18 years. Participants were excluded from the study if they met one of the following criteria: (a) regular consumption of alcohol (≥3 glasses daily for at least 3 weeks), use of cannabis (≥1 a week) or other illegal drugs [30], (b) suicidal thoughts or intentions within the last two weeks, diagnosis for (c) epilepsy, and (d) schizophrenia or acute psychosis. Other comorbid physical illnesses or mental disorders, shift work, sleep medication, and other psychotropic medications were accepted, though participants were asked to take their medication as prescribed and not to change dosage or frequency during the duration of their study participation. Participants were not financially compensated for their participation.

### Procedures

**Figure 1.**
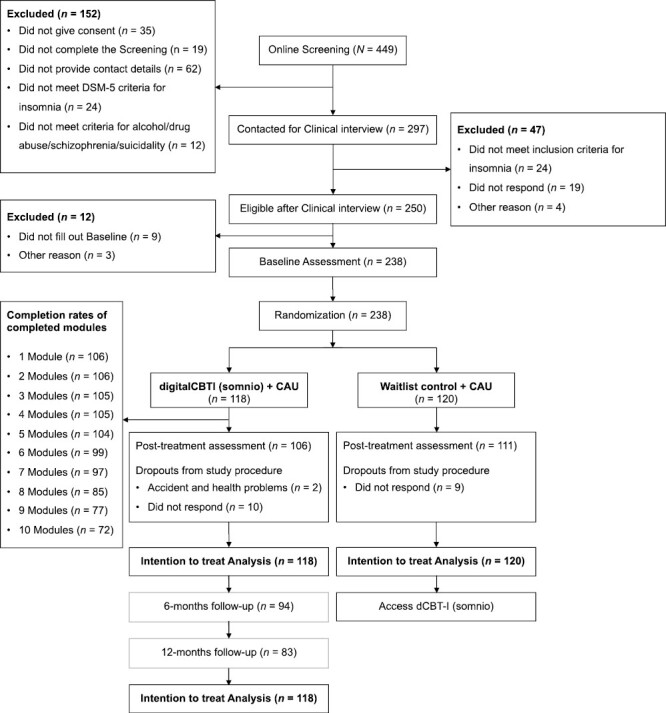
Flow chart showing participant selection, dropout, flow, and attrition through the intervention (assessed via self-report).

After reading the participation information sheet and giving their consent, participants completed a brief initial online screening (SoSci Survey GmbH), which examined the exclusion criteria (a–d), using a dichotomous response format (criterion present yes or no). Suitable participants were then contacted for a diagnostic telephone interview. Clinical interviews were conducted by supervised psychology students shortly before their master’s degree and by a clinical psychologist. During the clinical interview, participants were evaluated for insomnia, anxiety disorders, and mood disorders utilizing the open access version of the structured clinical interview for diagnosing mental disorders (DIPS-OA) according to DSM-5, Axis I [31]. Furthermore, they were questioned about suicidal thoughts using the suicide item of the Beck Depression Inventory [32], the intake of sleep medication, or any other medication. Eligible participants were then asked to complete the baseline assessment online. Upon completion of baseline assessments, they were randomly allocated to either the intervention group or the control group. Participants in the intervention group received access to dCBT-I for 8-weeks with the instruction to start immediately. Eight weeks post-randomization, both groups were instructed to complete the posttreatment assessment online. Participants in the control group were informed that they will receive access to dCBT-I upon completion of their post-intervention assessment and their study participation ended. Participants in the dCBT-I group were contacted for a brief telephone interview to discuss their personal experiences with the dCBT-I intervention. As part of the interview, they were explicitly asked about adverse events or side effects related to the intervention. Since the WLC did not receive an additional intervention, they were not asked about intervention-related side effects. However, all-participants were instructed to contact a member of the study team if they experienced any adverse events (e.g. hospitalization). The dCBT-I group was contacted for additional online follow-up measurements at 6- and 12-months post-randomization (see Figure 1). If assessments were not completed as instructed, participants were reminded four times by email or telephone.

### Randomization and masking

Participants were randomly assigned to either a dCBT-I intervention or a waitlist control group using a 1:1 randomization sequence with no stratification factors. The randomization sequence was kept by recruiting members of the study team who were unaware of previous allocations and assigned participants strictly in the temporal order of baseline completion, on which the study team had no influence (participants decided when to complete the baseline assessment online). Due to the nature of a behavioral intervention, participants were not blinded to the intervention and were aware of their group allocation. All-participants were informed that the study was setup to test the effectiveness of the digital intervention. Data was blinded before analysis to mask group allocation.

### The digital sleep intervention

Participants assigned to the intervention group were instructed to complete the dCBT-I intervention *somnio* (mementor DE GmbH), which was also available through practitioner referral in Germany at the time of the study. It comprises 10 core modules, each taking 5–25 min. The dCBT-I intervention *somnio* is fully automated and delivered by an interactive and animated avatar called “Albert” and can be accessed through a web browser or mobile app. New content is unlocked successively upon completion of previous modules and sleep diary entries. In addition, subsequent modules offer relapse prevention and help to consolidate previously learned content. Within the application, participants were advised to complete two to three modules per week. There was no homework besides following the instructions for stimulus control, sleep restriction therapy, sleep hygiene, and filling out the digital sleep diary. See Table 1 for an overview of the modules.

**Table 1.** Overview and description of dCBT-I modules during the intervention period

Module	Description
1.Introduction	Individual goals for sleep improvements are established and an overview of the intervention is given.
2.Sleep journal	The user is instructed to complete the sleep diary throughout the intervention period. Entries are used to evaluate progress and tailor the treatment to the individual.
3.Sleep knowledge	The user receives basic information about sleep regulation as part of psychoeducation.
4.Practical exercise	Previously acquired knowledge is applied and consolidated using practical examples. Dysfunctional beliefs are questioned.
5.Cycle of insomnia	Development of a disorder-specific explanation model: potential causes and perpetuating factors of insomnia are explained. An individual insomnia cycle visualizes the sleep difficulties of the user.
6.Sleeping^*^ hours	This module explains the principles of sleep restriction therapy and gives recommendations for an optimal sleep window. The sleep window is generated based on the average sleep duration of the last 7 days (minimum = 6 h). Weekly sleep window adjustment: sleep efficiency ≥ 85% => +15 min; sleep efficiency ≤ 80% => –15 min.
7.Relaxation	An introduction to progressive muscle relaxation (PMR) according to Jacobson is given and practiced.
8.Sleep behavior	In this module, users learn about stimulus control therapy and sleep hygiene.
9.Thoughts	Using various case examples, the participants’ individual dysfunctional thoughts on sleep-related issues are identified and questioned.
10.Everyday decisions	Typical everyday situations are presented to address safety behavior and its influence on sleep.
11.Closing session	Users are provided with a final comprehensive overview. They can test their acquired knowledge in the form of a quiz.
12.Aftercare	The progression of relevant sleep parameters and insomnia symptoms is assessed at regular intervals.

dCBT-I, digital cognitive behavioral therapy for insomnia.

^*^Users with bipolar disorder skip this module.

### Measurements

#### Primary Outcome.

##### Insomnia severity.

Self-reported insomnia severity was measured with the Insomnia Severity Index (ISI) [33]. The ISI asks about the sleep pattern of the previous two weeks and consists of seven items that are rated on a 5-point Likert scale from 0 (*not at all*) to 4 (*extremely*). Higher scores indicate greater insomnia severity. Items are summed up to a total score ranging from 0 to 28. The ISI reveals excellent internal consistency (α ≥ .90) [34, 35].

#### Secondary Outcomes.

##### Fatigue.

Fatigue was measured with the Fatigue Severity Scale (FSS) [36]. It consists of nine items, rated on a 7-point Likert scale ranging from 1 (*strong disagreement*) to 7 (*strong agreement*). Using the mean value, it assesses the perceived severity of fatigue symptoms during the past week. Higher values indicate greater fatigue. The FSS has shown good psychometric properties in a German-speaking sample and is one of the most widely used questionnaires for assessing daytime fatigue [37].

##### Daytime Sleepiness.

The Epworth Sleepiness Scale (ESS) [38, 39] is used to evaluate perceived daytime sleepiness. On a 4-point Likert scale ranging from 0 (n*o chance of dozing*) to 3 (*high chance of dozing*), the probability of falling asleep in eight everyday situations is asked retrospectively. The sum score of all-items results in a total score between 0 and 24, whereby a higher value indicates higher daytime sleepiness. The ESS is a validated and widely used instrument for the assessment of self-reported daytime sleepiness [38, 39].

##### Dysfunctional Beliefs and Attitudes about Sleep.

The short version of the Dysfunctional Beliefs and Attitudes About Sleep Scale (DBAS-16) [40, 41] identifies maladaptive sleep-related cognitions. The inventory comprises 16 items on an 11-point Likert scale from 0 (*strongly disagree*) to 10 (*strongly agree*). The DBAS-16 is a valid instrument that reliably detects dysfunctional beliefs and attitudes [42].

##### Well-Being.

The World Health Organisation-Five Well-being Index (WHO-5) [43, 44], measures mental well-being. It consists of five items on a 6-point Likert scale from 0 (*none of the time*) to 5 (*all of the time*). Scores are summed up to build a total score that ranges from 0 to 25 with higher scores indicating better well-being. Scores below 13 represent poor well-being and are indicative of depression [43]. The WHO-5 shows reliable psychometric properties for screening depression and assessing self-reported psychological well-being [45].

##### Quality of Life.

Quality of life was measured using the brief version of the WHO Quality of Life questionnaire (WHOQOL-BREF) [46]. The self-reported questionnaire consists of 26 items on a 5-point Likert scale. It distinguishes between four domain scores assessing the quality of life: physical health, psychological health, social relationships, and environment. Domain scores range from 4 to 20, with higher scores indicating better quality of life. The WHOQOL-BREF is a widely used and reliable measurement tool [46] and shows good validity in the German-speaking population [44].

##### Depressive Symptoms.

Depressive symptoms were quantified using the Allgemeine Depressionsskala (ADS-K) [47], the short form of the German version of the Center for Epidemiological Studies Depression Scale (CES-D) [48], which measures impairment due to depressive symptoms in the past week. It includes 15 items on a 4-point Likert scale ranging from 1 (*rarely, not at all*) to 4 (*most of the time, all the time*). The total score ranges from 0 to 45, with higher scores reflecting more depressive symptoms. Internal consistency has been confirmed in different samples and ranges from α = 0.88 to 0.95 [49]. A score of at least 18 has been shown to indicate a clinically relevant expression of depression, with a sensitivity of 89.7% and a specificity of 86.9% [50].

##### Anxiety Symptoms.

The trait version of the State-Trait Anxiety Inventory (STAI-T) [51, 52] was used to measure anxiety symptomatology. It consists of 20 items, each with a 4-point Likert scale, ranging from 1 (*almost never*) to 4 (*almost always*). Items are added up to a total score, which ranges from 20 to 80, with higher scores indicating higher trait anxiety. The trait anxiety subscale has shown good to excellent internal consistency (α = 0.89–0.91) [53]. A sum score at least 46 indicates the presence of clinically relevant anxiety symptoms [54].

##### Dream Recall Frequency.

Dream recall frequency was assessed using the Mannheim Dream Questionnaire (MADRE) [55]. The MADRE is a self-reported questionnaire that measures various dream-related aspects using 21 items. On a 7-point Likert scale, from 0 (*never*) to 6 (*almost every morning*), participants indicate how often they remembered their dreams in the *“past months.”* The MADRE has shown good psychometric properties in a German-speaking population [55].

##### Nightmare Frequency.

The Nightmare Distress Questionnaire (NDQ) [56] was used to investigate the nightmare frequency of the past four weeks. Participants answer the question “*How many nightmares have you had in the past four weeks?*” in open response format [57].

##### Adherence And Treatment Satisfaction.

At posttreatment assessment, participants in the dCBT-I group were asked how many modules they had completed using a multiple-choice item with given answer options from 0 to 10 modules, to operationalize adherence. Additionally, three 5-point Likert scales captured participants’ satisfaction with the intervention (“How satisfied have you been?” [1 = *very dissatisfied*, 2 = *dissatisfied*, 3 = *neutral*, 4 = *satisfied*, 5 = *very satisfied*]; “Did the intervention meet your expectations?” [1 = *not at all*, 2 = *a little*, 3 = *partly*, 4 = *mostly*, 5 = *completely*]) and on the accuracy with which participants implemented dCBT-I (“How conscientiously did you complete the modules?” [1 = *very conscientiously*, 2 = *conscientiously*, 3 = *neutral*, 4 = *not conscientiously*, 5 = *not at all conscientiously*]).

##### Sleep Diary.

The sleep diary is an integrated function of *somnio* and is comprised of a morning and an evening log. The morning log asks about last night’s sleep parameters, i.e. sleep onset latency (SOL = *[“sleep onset” – “lights off”]*), wake after sleep onset (WASO = *[“time awake after sleep onset”] + [“rise time” – “wake-up time”]*), total sleep time (TST = *[“wake-up time” – “sleep onset” – “time awake after sleep onset”]*), time in bed (TIB = *[rise time—bedtime]*) and sleep efficiency (SE = *[TST/TIB* × 100*]*). Furthermore, overall sleep quality was assessed using a visual analogue scale ranging from 0 (*bad*) to 100 (*good*).

### Statistical analyses

In the original protocol, we calculated our power on an analysis of variance (ANOVA) with repeated measures. It yielded a sample size of *n *= 124. The sample size was calculated using G*Power [58] with α = 0.05 and power of 1 – β = 0.9, to detect medium effect sizes (*d *= 0.5), which we estimated conservatively in a heterogeneous study sample [13]. Given an expected attrition rate of 28% [59], we initially sought to recruit *n* = 79 participants per group. However, in response to recent recommendations for statistical analyses of two-armed randomized pre-post designs with one posttreatment assessment (c.f [60].), we decided it was more appropriate to employ an analysis of covariance (ANCOVA), considering the baseline value of the primary outcome. The updated power analysis for an ANCOVA with the same parameters and 28% dropout yielded a total sample size of *N* = 220. No further methodological changes were made in the process of the trial.

All-analyses were conducted using SPSS.25 (IBM). Consistent with CONSORT guidelines, all-data from all randomized participants were analyzed according to the intention-to-treat approach [61]. Missing data were searched for systematic patterns and, when confirming the missing at random (MAR) condition, reconstructed with multiple imputations [62] using the linear regression model, with pooled data from five imputed datasets. No adjustment was made for multiple testing for the analyses performed on the primary and secondary outcomes as these were prespecified [63]. Descriptive statistics are presented by unadjusted means (*M*) and standard deviations (SD) for continuous outcomes, and frequencies for binary outcomes. Analysis of covariance (ANCOVA), adjusting for the baseline value of the respective outcome, was used to determine between-group differences at 8-weeks post-randomization.

Responder (ISI change ≥ 8) and remission rates (ISI total score < 8) were calculated for the primary outcome (ISI) to determine clinical significance [33, 34]. Between-group differences of dichotomous outcomes were analyzed using Pearson chi-squared tests and effect sizes were quantified using phi. Between-group effect sizes of continuous outcomes were calculated by dividing the adjusted mean difference by the pooled standard deviation of both groups at baseline [64]. Linear-mixed models (LMMs) were fitted for continuous sleep diary and follow-up data in the intervention group, with fixed effects for time points (week 1–week 8 for sleep diary data and baseline, week 8, 24, and 48 for long-term follow-up outcomes). Within-group effect sizes were calculated by dividing the mean difference by the standard deviation of the change [64]. For sleep diary data, outcomes at week one and week 8 were included as the response. A participant-specific random intercept was embedded to account for repeated measures and their non-independence. The covariance structure for random effects, here participants, was set to variance components (VC) as this is the default for mixed models with one random effect [65].

## Results

### Participant and clinical characteristics

Overall, *n* = 449 participants completed the screening between February and May 2021 and *n* = 297 fulfilled all-requirements for further participation. Recruitment was stopped as soon as 220 participants were randomized but spillovers were included for ethical reasons. After the clinical interview, *n* = 250 participants were deemed eligible, of which *n* = 238 completed baseline assessment, were randomized and included in all subsequent analyses. All-participants randomized to the intervention (*n* = 118) were followed-up at 6- and 12-months post-randomization and included in all-analyses. The follow-up assessments took place from October 2021 to February 2022 (6-months follow-up) and April 2022 to September 2022 (12-month follow-up). Most of the participants had a university degree (*n* = 136, 57.1%) or completed an apprenticeship (*n* = 52, 21.8%). Demographics and baseline characteristics are depicted in Table 2.

**Table 2.** Participant baseline characteristics

	dCBT-I + CAU	WLC + CAU	All
	*n* = 118	*n* = 120	*n *= 238
Baseline characteristics			
Age, years, *M (SD)*	44.27 (14.25)	43.20 (13.57)	43.73 (13.90)
Female, *n* (%)	82 (69.5)	79 (65.8)	161 (67.6)
Symptom duration, years, *M (SD)*	8.91 (10.25)	6.20(7.74)	7.55 (9.15)
Distinct trigger that caused symptoms, *n* (%)	23 (19.5)	29 (24.2)	52 (21.8)
Shared bedroom, *n* (%)	57 (48.3)	45 (37.5)	102 (42.9)
Shift work, *n* (%)	3 (2.5)	6 (5.0)	9 (3.8)
Children disrupting sleep, *n* (%)	11 (9.3)	10 (8.3)	21 (8.8)
Psychotherapy			
Current, *n* (%)	23 (19.5)	17 (14.2)	40 (16.8)
Former, *n* (%)	31 (26.3)	52 (43.3)	83 (34.9)
CNS medication, *n* (%)	2 (1.7)	3 (2.5)	5 (2.1)
Sleep medication, *n* (%)	49 (41.5)	44 (36.7)	93 (39.1)
Other medication, *n* (%)	13 (11.0)	15 (12.5)	28 (11.8)
Comorbidities			
Total comorbidities^*^, *n* (%)	86 (72.9)	88 (73.3)	174 (73.1)
Physical illnesses, *n* (%)	50 (42.4)	47 (39.2)	97 (40.8)
Psychological diagnoses			
Current, *n* (%)	14 (11.9)	13 (10.8)	27 (11.3)
In the past, *n* (%)	22 (18.6)	34 (28.3)	56 (23.5)
Sleepwalking, *n* (%)	4 (3.4)	1 (0.8)	5 (2.1)
Narcolepsy, *n* (%)	0 (0.0)	0 (0.0)	0 (0.0)
Pavor nocturnus, *n* (%)	2 (1.7)	0 (0.0)	2 (0.8)
Bruxism, *n* (%)	25 (21.2)	24 (20.0)	49 (20.6)
Nightmare disorder, *n* (%)	3 (2.5)	1 (0.8)	4 (1.7)
Restless legs, *n* (%)	4 (3.4)	4 (3.3)	8 (3.4)
Obstructive sleep apnea, *n (%)*	6 (5.1)	5 (4.2)	11 (4.6)
Outcomes at baseline			
ISI, *M (SD)*	17.63 (3.90)	17.06 (3.42)	17.34 (3.67)
FSS, *M (SD)*	4.57 (1.28)	4.68 (1.09)	4.62 (1.19)
ESS, *M (SD)*	8.18 (4.28)	7.15 (4.13)	7.66 (4.23)
DBAS-16, *M (SD)*	5.40 (1.35)	5.53 (1.28)	5.47 (1.31)
WHO-5, *M (SD)*	10.31 (4.11)	9.72 (4.03)	10.01 (4.07)
WHOQOL-BREF			
Physical health, *M (SD)*	14.09 (2.29)	14.07 (2.29)	14.08 (2.29)
Psychological health, *M (SD)*	13.92 (2.56)	13.48 (2.42)	13.69 (2.50)
Social relationship, *M (SD)*	14.29 (3.10)	13.94 (3.38)	14.12 (3.24)
Environment, *M (SD)*	16.48 (2.10)	16.48 (2.02)	16.48 (2.06)
ADS-K, *M (SD)*	15.40 (6.86)	15.45 (6.90)	15.42 (6.86)
STAI-T, *M (SD)*	45.28 (10.21)	46.19 (10.28)	45.74 (10.23)
Dream recall frequency, *M (SD)*	3.47 (1.72)	3.28 (1.77)	3.38 (1.75)
Nightmare frequency, *M (SD)*	2.02 (5.28)	1.28 (3.06)	1.64 (4.31)

dCBT-I, digital cognitive behavioral therapy for insomnia; CAU, care as usual; WLC, waitlist control; ISI, Insomnia Severity Index; FSS, Fatigue Severity Scale, ESS, Epworth Sleepiness Scale; DBAS-16, Dysfunctional Beliefs and Attitudes About Sleep Scale; WHO-5, World Health Organisation-Five Well-Being Index; WHOQOL-BREF, WHO Quality of Life questionnaire; ADS-K, general depression scale; STAI-T, State-Trait Anxiety Inventory (trait anxiety questions).

^*^Number of participants with at least 1 comorbidity.

### Effects on the primary outcome

**Figure 2.**
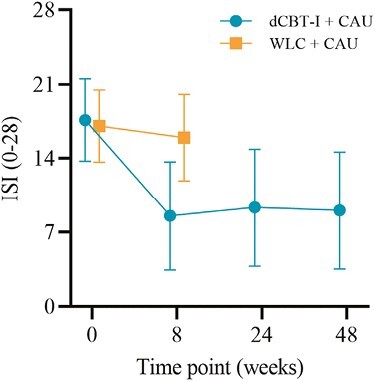
Changes in primary outcome and insomnia severity, across both groups and all-assessments. Unadjusted means (±SD) are presented for both groups at baseline and posttreatment, and for the intervention group at 6- and 12-month follow-ups.

The results of the statistical analyses are presented in Table 3. Regarding self-reported insomnia severity (ISI), a large between-group effect was found in favor of the intervention group, *F*(1, 435.46) = 182.76, *p* < .001, *d* = –2.08 (see Figure 2).

**Table 3.** Between-group effects of dCBT-I and WLC on all-outcomes

	dCBT-I + CAU		WLC + CAU		Diff_adj_	95% CI_pooled_		*P*	ES
	*n* = 106		*n *= 111						
	*M*	SD	*M*	SD					
ISI	8.56	5.09	15.96	4.09	–7.60	–8.67	–6.54	**<.001**	–2.08
FSS	3.44	1.33	4.77	1.01	–1.21	–1.47	–0.97	**<.001**	–1.02
ESS	7.32	4.30	7.68	4.26	–1.08	–1.74	–0.41	**.003**	–0.26
DBAS-16	3.90	1.52	5.56	1.33	–1.54	–1.83	–1.26	**<.001**	–1.17
WHO-5	13.67	4.78	10.18	4.49	2.76	1.86	3.70	**<.001**	0.68
WHOQOL-BREF									
Physical health	15.80	2.53	14.06	2.23	1.52	1.10	1.98	**<.001**	0.66
Psychological health	14.68	2.53	13.62	2.44	0.48	0.11	0.87	**.016**	0.19
Social relationships	14.60	3.28	13.63	3.18	0.47	–0.09	1.03	.121	0.15
Environment	16.82	2.06	16.38	2.02	0.30	–0.01	0.64	.090	0.15
ADS-K	10.23	6.86	16.10	7.44	–5.48	–6.96	–4.02	**<.001**	–0.80
STAI-T	39.78	10.38	46.93	10.05	–5.73	–7.37	–4.16	**<.001**	–0.56
Dream recall frequency	3.21	1.86	3.32	1.79	–0.32	–0.66	0.08	.257	–0.18
Nightmare frequency	1.43	4.86	1.13	3.06	0.28	0.97	–0.59	.681	–0.07

dCBT-I, digital cognitive behavioral therapy for insomnia; *M* and *SD* refer to unadjusted means and standard deviations, respectively; Diff_adj_, adjusted mean difference derived from fitted ANCOVAs; 95% CI_pooled_, 95% confidence interval of the pooled adjusted mean difference; ES, between-group effect size (Cohen’s *d*).

The superiority of the dCBT-I group was also reflected in responder and remission rates. At posttreatment assessment, 63.6% (*n* = 75) of participants in the dCBT-I group were classified as responders, compared to 5.8% (*n* = 7) of participants in the control group, χ² (1, *n *= 217) = 95.80, *p* < .001, φ = –.66. Additionally, 40.7% of participants (*n* = 48) in the dCBT-I group achieved remission, compared to 1.7% of participants (*n* = 2) in the control group, χ²(1, *n *= 217) = 57.81, *p* < .001, φ = –0.52.

### Effects on secondary outcomes

#### Fatigue.

A large between-group difference was found in favor of the dCBT-I group, *F*(1, 3090.42) = 89.362, *p* < .001, *d* = –1.02. Participants in the dCBT-I group reported lower scores on the FSS scale, compared to the control group.

#### Daytime sleepiness.

The ANCOVA revealed a small between-group effect on the ESS and in favor of dCBT-I, *F*(1, 174.87) = 9.03, *p* = .003, *d* = –0.26.

#### Dysfunctional beliefs and attitudes about sleep.

**Figure 3.**
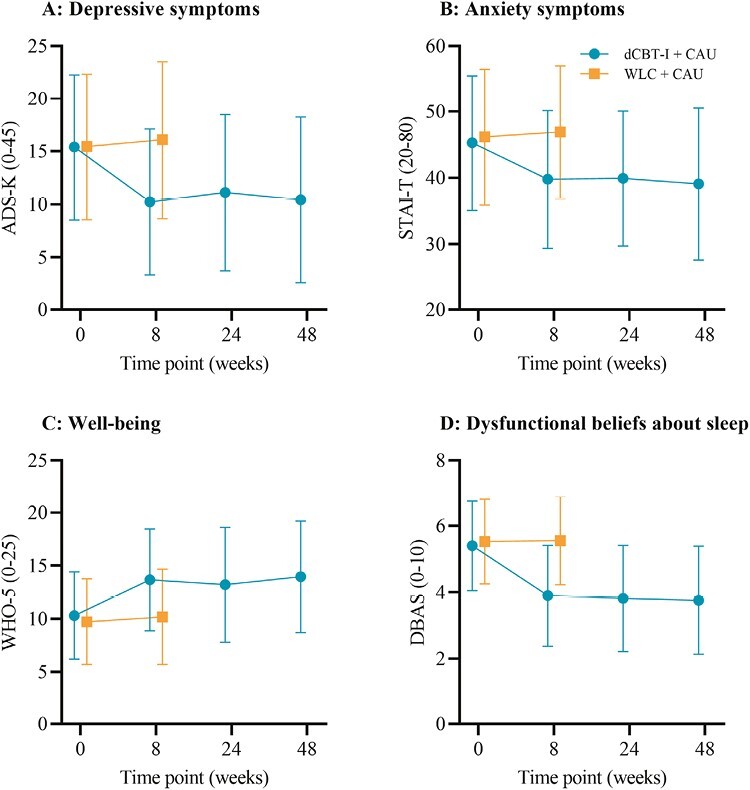
Changes in secondary outcomes: (A) depressive symptoms, (B) anxiety symptoms, (C) Well-being, and (D) Dysfunctional beliefs about sleep, across both groups and all-assessments. Unadjusted means (±SD) are presented for both groups at baseline and posttreatment, and for the intervention group at 6- and 12-month follow-ups.

A similar pattern emerged for dysfunctional beliefs and attitudes about sleep. Participants in the dCBT-I group reported improvements on the DBAS, when compared to the control group, *F*(1, 199.61) = 101.31, *p* < .001, *d* = –1.17 (see Figure 3D).

#### Well-being.

A medium between-group effect size was found on the WHO-5 questionnaire, again favoring the dCBT-I group when compared to the control group, *F*(1, 9074.29) = 34.56, *p* < .001, *d* = 0.68 (see Figure 3C).

#### Quality of Life.

Quality of life was analyzed by domain, revealing medium-sized effects for *physical health* in favor of the dCBT-I group, *F*(1, 86.64) = 37.21, *p* < .001, *d* = 0.66, and small effect sizes on the domain *psychological health*, also in favor of the dCBT-I group, *F*(1, 1235.15) = 5.77, *p* = 0.164, *d* = 0.19. In contrast, no between-group differences were detected in the domains *social relationships* and *environment*, *F*(1, 208.08) = 2.42, *p* = .121, *d *= 0.15 and *F*(1, 196.90) = 2.91, *p* = .090, *d* = 0.15, respectively.

#### Depressive symptoms.

Regarding depressive symptoms, a large between-group effect was found *F*(1, 950.75) = 51.30, *p* < .001, *d* = –0.80. Participants reported fewer depressive symptoms after dCBT-I compared to the control group (see Figure 3A).

#### Anxiety symptoms.

A medium between-group effect with superiority of the dCBT-I group was found for anxiety symptoms *F*(1, 7565.44) = 49, *p* < .001, *d* = –0.56 (see Figure 3B).

#### Dream recall frequency.

No difference in dream recall frequency was found between groups at posttreatment assessment *F*(1, 94.70) = 2.35, *p* = .257, *d* = –0.18.

#### Nightmare frequency.

With regard to nightmare frequency, no between-group effect was detected *F*(1, 211.03) = 0.16, *p* = .681, *d* = –0.07.

#### Adherence, treatment satisfaction and adverse events.

On average, participants completed *n* = 9.02 modules during the intervention period. Of all-participants who received dCBT-I, 61.02% (*n* = 72) stated that they completed all 10 core modules, and 5.93% (*n* = 7) of participants completed five modules or less. Most participants stated that they had worked on the modules with at least a *predominant* degree of conscientiousness (*n* = 95, 80.51%). Seventy-two participants (61.02%) were *satisfied* or *very satisfied* with the dCBT-I intervention. Another *n* = 22 participants (18.64%) were neutral towards the intervention. For *n *= 87 of the participants (73.73%), their expectations regarding the CBT-I intervention were at least partially fulfilled. Besides eight (6.78%) individual reports of daytime fatigue and/or feeling distressed, especially during bedtime restriction, no severe side effects or adverse events have been reported from the *n* = 118 participants, which were randomized to the intervention group.

#### Effects on sleep diary parameters.

From week one to week eight, SOL and WASO decreased on average by 28 min (*p < *.001) and 64 min (*p* < .001), respectively. Total sleep time increased by an average of 25 min (*p* < .001). Sleep efficiency increased by approximately 16% (*p* < .001). See Table 4 for the results of the mixed-effect model.

**Table 4.** Effects of dCBT-I on sleep diary parameters of the intervention group

	Week 1		Week 8		Diff_adj_	95% CI	*P*	ES
	*n* = 95		*n* = 95					
	*M*	SD	*M*	SD				
SOL (min)	49.09	53.95	15.94	22.85	–28.54	–32.81, –24.27	**<.001**	–1.34
WASO (min)	111.69	102.17	45.12	41.41	–64.22	–71.50, –56.95	**<.001**	–1.78
TST (min)	354.59	109.09	381.50	82.02	25.34	15.37, 35.30	**<.001**	0.51
SE (%)	68.76	18.98	86.10	11.64	16.09	14.53, 17.64	**<.001**	2.08

dCBT-I, digital cognitive behavioral therapy for insomnia; SOL, sleep onset latency; WASO, wake after sleep onset; TST, total sleep time; SE, sleep efficiency; Diff_adj_, adjusted mean difference derived from linear mixed models; 95% CI, 95% confidence interval of the adjusted mean difference; ES, within-group effect size (Cohen’s *d*). *M* and SD refer to unadjusted means and standard deviations, respectively.

#### Long-term effects.

Within-group comparisons were conducted at 6- and 12-month follow-ups in the intervention group and revealed large effect sizes for insomnia severity at both time points in comparison to baseline values, indicating that treatment effects were stable over time (*d* = –1.39 and *d* = –1.26). Sustained treatment effects were also observed for measurements of fatigue, daytime sleepiness, dysfunctional beliefs about sleep and well-being (*d*s ranging from 0.42 to 1.65). Again, assessments of quality of life revealed treatment effects for the physical and psychological health domain (*d*s ranging from 0.18 to 0.66), but not for the domains of social relationships and environment (*d*s ranging from 0.00 to 0.16). Stable long-term effects were also observed with regard to measures of depression and anxiety symptoms (*d*s ranging from –0.54 to –0.60). In contrast, but in line with posttreatment group comparisons, we observed no long-term effects for dream recall frequency or nightmare frequency (*d*s ranging from –0.03 to –0.18).

See Table 5 for an overview of all long-term results in the intervention group.

**Table 5.** Within-group effects of dCBT-I at 2-, 6- and 12-months post-randomization

	Time (month)		*n*	*M*	SD	Diff_adj_	95% CI		*P*	ES
ISI	0		118	17.63	3.90					
		2	106	8.56	5.09	–9.05	–8.06	–10.03	**<.001**	–1.68
		6	94	9.35	5.53	–8.14	–7.08	–9.21	**<.001**	–1.39
		12	83	9.06	5.53	–8.07	–6.91	–9.24	**<.001**	–1.26
FSS	0		118	4.57	1.28					
		2	106	3.44	1.33	–1.01	–0.85	–1.35	**<.001**	–0.80
		6	94	3.25	1.37	–1.30	–1.04	–1.56	**<.001**	–0.91
		12	83	3.42	1.48	–1.11	–0.81	–1.40	**<.001**	–0.69
ESS	0		118	8.18	4.28					
		2	106	7.32	4.30	–0.87	–0.27	–1.46	**.005**	–0.26
		6	94	6.93	3.96	–1.32	–0.74	–1.89	**<.001**	–0.42
		12	83	3.27	4.14	–5.04	–4.48	–5.59	**<.001**	–1.65
DBAS-16	0		118	5.40	1.35					
		2	106	3.90	1.52	–1.49	–1.22	–1.75	**<.001**	–1.02
		6	94	3.82	1.60	–1.54	–1.28	–1.79	**<.001**	–1.10
		12	83	3.76	1.63	–1.51	–1.22	–1.79	**<.001**	–0.96
WHO-5	0		118	10.31	4.11					
		2	106	13.67	4.78	3.26	4.02	2.50	**<.001**	0.79
		6	94	13.21	5.40	2.59	3.50	1.68	**<.001**	0.52
		12	83	13.96	5.26	3.27	4.18	2.35	**<.001**	0.65
WHOQOL-BREF										
Physical health	0		118	14.09	2.29					
		2	106	15.80	2.53	1.60	1.97	1.24	**<.001**	0.80
		6	95	15.70	2.55	1.31	1.67	0.95	**<.001**	0.66
		12	83	15.65	2.63	1.26	1.71	0.81	**<.001**	0.51
Psychological health	0		118	13.92	2.56					
		2	106	14.68	2.53	0.57	0.87	0.27	**<.001**	0.35
		6	95	14.58	2.50	0.44	0.75	0.14	**.005**	0.27
		12	83	14.63	2.86	0.43	0.86	0.01	**.047**	0.18
Social relationships	0		118	14.29	3.10					
		2	106	14.60	3.28	0.07	0.52	–0.37	.743	0.03
		6	95	14.09	3.19	–0.35	0.15	–0.84	.170	–0.13
		12	83	14.52	3.10	–0.01	0.58	–0.60	.977	0.00
Environment	0		118	16.48	2.10					
		2	106	16.82	2.06	0.25	0.52	–0.03	.078	0.16
		6	95	16.77	2.30	0.09	0.41	–0.23	.582	0.05
		12	83	16.74	2.14	0.01	0.32	–0.30	.948	0.01
ADS-K	0		118	15.40	6.86					
		2	106	10.23	6.86	–5.00	–3.76	–6.23	**<.001**	–0.74
		6	94	11.11	7.36	–4.05	–2.79	–5.30	**<.001**	–0.59
		12	83	10.43	7.80	–4.35	–2.86	–5.81	**<.001**	–0.54
STAI-T	0		118	45.28	10.21					
		2	106	39.78	10.38	–5.24	–3.75	–6.73	**<.001**	–0.64
		6	94	39.91	10.15	–4.97	–3.44	–6.49	**<.001**	–0.60
		12	83	39.07	11.46	–5.09	–3.44	–6.75	**<.001**	–0.56
Dream recall frequency	0		118	3.47	1.72					
		2	106	3.21	1.86	–0.21	0.09	–0.51	.172	–0.13
		6	95	3.25	1.83	–0.09	0.17	–0.34	.515	–0.06
		12	83	3.04	1.76	–0.31	0.01	–0.62	.058	–0.18
Nightmare frequency	0		118	2.02	5.28					
		2	106	1.43	4.86	–0.56	0.14	–1.26	.115	–0.15
		6	95	1.33	4.05	–0.68	0.27	–1.62	.159	–0.13
		12	83	1.93	6.63	–0.24	1.01	–1.49	.702	–0.04

ISI, Insomnia Severity Index; FSS, Fatigue Severity Scale, ESS, Epworth Sleepiness Scale; DBAS-16, Dysfunctional Beliefs and Attitudes About Sleep Scale; WHO-5, World Health Organisation-Five Well-Being Index; WHOQOL-BREF, WHO Quality of Life Questionnaire; Diff_adj_, adjusted mean difference derived from linear mixed models; 95% CI, 95% confidence interval of the adjusted mean difference; ES, within-group effect size (Cohen’s *d*). *M* and SD refer to unadjusted means and standard deviations, respectively.

## Discussion

The present study investigated the effects of digital cognitive behavioral therapy for insomnia (dCBT-I) in comparison to a waitlist control group (WLC). The study setup aimed to recruit a heterogenous insomnia population by setting very limited exclusion criteria. The results of this study showed that low-intensity, digital sleep intervention improved clinical outcomes in addition to usual care in people with insomnia, many of whom suffer with at least one comorbid mental disorder or physical illness. We found large effects of the intervention on insomnia severity and fatigue, and medium-to-large effects on well-being, physical health-related quality of life, symptoms of depression, and anxiety.

Improvements during the dCBT-I intervention in sleep continuity and large between-group effects on dysfunctional beliefs about sleep support CBT-I mechanism of action. These findings largely support our hypotheses and are in accordance with previous studies that investigated the effects of dCBT-I on clinical and sleep-related outcomes [7–9]. Despite the heterogeneity of the sample and prevalence of comorbid conditions, the proportion of participants in the dCBT-I group who were considered treatment responders was found to be high, when compared to a previous trial using the same intervention in a more homogenous study [66]. This is in line with the current state of research showing that dCBT-I is highly effective across different clinical groups of participants (for an overview see Luik and Espie [67]). Large dCBT-I treatment effects may also be explained by high treatment satisfaction and strong adherence rates, which were comparatively high in this study with 88.14% completing at least half of the modules [59]. High treatment satisfaction and strong adherence rates have previously been hypothesized as predictors of CBT-I treatment outcomes [59, 68] and may explain the large treatment effects observed (e.g. *d* = –2.08 with regard to ISI).

Still, a significant number of participants in this trial did not complete all-modules. And although the use of technology may not be equivalent to treatment adherence, it indicates user interest in the intervention. Indeed, a higher dropout rate was observed in module six, when sleep restriction therapy was introduced. An observation that was confirmed by our posttreatment interviews, that a total of 19 participants (16.1%) stated that they had selectively discontinued the sleep restriction therapy module or had subsequently stopped the entire intervention. Furthermore, participants mentioned that modules were forgotten due to a lack of reminders and that it was therefore not possible to complete all-modules in 8-weeks. Although it is possible to set reminders within the application, an automatic reminder function could counteract poor adherence due to forgetting.

Despite the fact that the participants’ well-being improved, scores posttreatment were still below the population average [44]. This could be explained by the high percentage of comorbidities [69] or, potentially, by the time of recruitment which took place during the lockdown phases in Germany due to the Corona SARS-CoV-2 pandemic, which was associated with lower well-being [70] and may have impacted the results.

The effects on daytime sleepiness and mental health-related quality of life were smaller in magnitude and absent for domain social relationships as well as for domain environment. While effects on daytime sleepiness are typically expected in the small range [71], effects on quality of life are mixed and seem to be depending on the presence of comorbid conditions (i.e. smaller effects for patients with insomnia and a comorbid disorder) and the choice of quality of life measure [72]. Yet, our study revealed improvements in symptoms of anxiety and depression, compared to control and in the long-term, which underscores the potential of insomnia interventions in the treatment of emotion-regulation disorders [73]. We found no group differences for dream recall frequency and nightmare frequency. These null findings may reflect true effects or could be due to floor effects since both frequencies were already low at baseline compared to other samples with insomnia or other sleep disorders [74].

Results of 6- and 12-month follow-up measurements in the intervention group indicated the stability of treatment effects, although small deteriorations were observed for insomnia severity (see Figure 2). These results are in line with results from a recent meta-analysis showing lasting treatment effects for CBT-I up to 1 year, despite small declines [9].

Intriguingly, for other measures an inverse pattern was observed (e.g. daytime sleepiness; 8-weeks *d* = –0.26, 6-months *d* = –0.42, 12-months *d* = –1.65), indicating that some treatment effects may also increase over time. Yet, the absence of a control group at long-term follow-up and the relaxation of population-wide lockdown measures (due to SARS-CoV-2) at the time of our 12-month assessment may limit the interpretation of those findings.

Despite promising results, a number of limitations need to be mentioned. First, a waitlist control group was used as the choice of comparator, potentially inflating effect sizes on self-reported outcomes due to awareness of group assignment (open-label). Consequently, treatment expectancy may have biased responses in the intervention group and dampened active health behavior in the control group [75]. However, the setup of this study aimed to reflect regular care, which, in Germany, does not involve sleep advice or any other health support besides medication [10], which was explicitly not restricted. Second, our recruitment was primarily online and may have favored participants with an affinity for internet-based products. Yet, conducting the study fully online allowed us to complete the study during an ongoing pandemic without any limitations. Third, we did not correct for multiple testing and performed explorative analysis on sleep diary outcomes. Consequently, the risk of type I error is evident [76]. However, we reported exact values and effect sizes to allow the reader to judge [77].

In this study, we showed that dCBT-I yields improvements beyond insomnia and sleep-related symptoms, enhancing overall well-being and daytime functioning in a heterogenous insomnia population. Results strongly support previous evidence for the use of digital formats to treat insomnia and, due to our limited exclusion criteria, and thus high external validity, allows generalizability to the insomnia population in Germany.

## Clinical Trial Information

Name: Effects of a digital Cognitive-Behavior Therapy for Insomnia (CBT-I) [German: Wirksamkeit einer digitalen kognitiv-behavioralen Psychotherapie zur Behandlung von Insomnie (KVT-I)].

URL: https://drks.de/search/en/trial/DRKS00024477

Registration number: DRKS00024477.
